# Overexpression of BRCA1 in Neural Stem Cells Enhances Cell Survival and Functional Recovery after Transplantation into Experimental Ischemic Stroke

**DOI:** 10.1155/2019/8739730

**Published:** 2019-04-03

**Authors:** Pengfei Xu, Xiaolei Shi, Xiaohao Zhang, Qian Liu, Yi Xie, Ye Hong, Juanji Li, Mengna Peng, Xinfeng Liu, Gelin Xu

**Affiliations:** ^1^Department of Neurology, Jinling Hospital, Medical School of Nanjing University, Nanjing, 210002 Jiangsu, China; ^2^University of British Columbia, Kinsmen Laboratory of Neurological Research, Vancouver, British Columbia, Canada; ^3^Department of Neurology, The First Affiliated Hospital, Yijishan Hospital of Wannan Medical College, Wuhu, 241001 Anhui, China; ^4^Department of Neurology, Jiangsu Provincial Second Chinese Medicine Hospital, The Second Affiliated Hospital of Nanjing University of Chinese Medicine, Nanjing, 210002 Jiangsu, China

## Abstract

Transplantation of neural stem cells (NSCs) is a promising therapy for ischemic stroke. However, the effectiveness of this approach is limited by grafted cell death. Breast cancer susceptibility protein 1 (BRCA1) could suppress apoptosis in neural progenitors and modulate oxidative stress in neurons. In this study, we found that BRCA1 was upregulated by oxygen-glucose deprivation/reoxygenation (OGD/R). Overexpression of BRCA1 in NSCs reduced cell apoptosis and oxidative stress after OGD/R insult. The molecule overexpression also stimulated cellular proliferation in OGD/R NSCs and increased the survival rate of grafted cells. Further, the transplantation of BRCA1-transfected NSCs into mice with ischemic stroke increased brain-derived neurotropic factor and nerve growth factor expression in the brain and elicited neurological function improvement. In addition, we found that RING finger domain and BRCT domain of BRCA1 could physically interact with p53 in NSCs. The cross talk between BRCA1 RING finger domain and p53 was responsible for p53 ubiquitination and degradation. Our findings indicate that modification with BRCA1 could enhance the efficacy of NSCs transplantation in ischemic stroke.

## 1. Introduction

Ischemic stroke is the second leading cause of death globally [1]. Stem cell transplantation is emerging as a viable therapy for this disease through its unique effects of trophic factor support, neural cell replacement, and endogenous brain repair process [2–4]. However, the majority of grafted cells do not survive after transplantation. It could be attributed to oxidative stress and subsequent inflammation during ischemia/reperfusion (I/R) [5–7]. This highly hinders the wide application of stem cell therapy in clinical practice. It is then essential to find suitable targets to ensure the credibility of stem cell therapy.

Breast cancer susceptibility protein 1 (BRCA1), known as a tumor suppressor, is expressed in neural precursor/stem cells (NPCs/NSCs) [8, 9]. We previously demonstrated that overexpression of BRCA1 attenuates neuronal oxidative stress [10]. And it is believed that oxidation balance is a vital factor for the survival of cells after transplantation [5]. BRCA1 ablation could induce p53-associated apoptotic pathways in NPCs [9, 11] and is responsible for embryonic cellular proliferation [12]. However, whether BRCA1 could rescue NSCs against I/R injury has not been clearly elucidated. In this study, we examined the role of BRCA1 in NSCs apoptosis and oxidative injury. Whether BRCA1 could improve grafted NSCs survival as well as neurological recovery will also be explored.

## 2. Materials and Methods

### 2.1. Animal and Cerebral I/R Model

Adult male C57BL/6J mice (20-25 g) and pregnant C57BL/6J mice were purchased from the Model Animal Research Center of Nanjing University and housed in a controlled environment (temperature: 24 ± 1°C; relative humidity: 50-60%) with a 12 h light/dark cycle. This study was approved by Jinling Hospital Research Ethics Committee and performed according to the National Institutes of Health Guide for the Care and Use of Laboratory Animals (NIH Publication no. 80-23 revised in 1996).

Transient focal cerebral ischemia was induced by middle cerebral artery occlusion (MCAO) surgery following previous methods [10, 13]. Briefly, mice were anesthetized with 2% isoflurane in O_2_ using a face mask. The right external carotid artery (ECA) and internal carotid artery (ICA) were dissected. To occlude the MCA, a 6-0 silicon-coated monofilament nylon suture (Beijing Cinontech Co., China) was introduced into the ICA through the ECA until mild resistance was felt. A decline of the regional cerebral blood flow ≥ 75% was considered successful occlusion as monitored by a laser Doppler flowmetry (PeriFlux 5010; Perimed AB, Sweden). After 90 min of occlusion, the monofilament was withdrawn for reperfusion. Body temperature was maintained at 37°C using a heating pad during the surgery.

### 2.2. NSCs Culture

NSCs were harvested from embryonic C57BL/6J mouse brain (E14) as previously described [14]. Briefly, cells were resuspended in a DMEM/F12 medium containing B27 (2%, Gibco, USA), EGF (20 ng/ml, BioLegend, USA), bFGF (20 ng/ml, BioLegend, USA), and ITSS (10 *μ*g/ml, Roche, Switzerland) and cultured in a cell incubator. The medium was changed every 3 d, and cells were passaged when the neurospheres grew to 50-100 *μ*m diameter. Cells were plated on poly-D-lysine and laminin precoated coverslips for further experiments.

### 2.3. Oxygen-Glucose Deprivation/Reoxygenation (OGD/R) Induction

NSCs OGD/R was induced according to previously described methods with modification [15]. In brief, OGD was carried out by replacing the cell culture medium with a glucose-free DMEM (Gibco, USA). Then, the culture was incubated in an anaerobic chamber at 37°C. After 4 h, the cells were returned to 5% CO_2_/95% air incubator with a normal NSC medium for reoxygenation.

### 2.4. Lentivirus Transfection and Intracerebral Transplantation

The entire mouse BRCA1 sequence (1-5438 bp) and RING finger domain deletion sequence (517-5438 bp) were cloned in pLV/EF1A/eGFP plasmid. And pLV/EF1A/eGFP-null plasmid was used as control vector. 293T cells were transfected with these vectors to obtain lentiviruses. Lentivirus-GFP (LV-GFP), lentivirus-mutant BRCA1-GFP (LV-BRCA1-mut), and lentivirus-wild-type BRCA1-GFP (LV-BRCA1) were further purified by centrifugation [10]. NSCs were transfected with LV-GFP, LV-BRCA1-mut, or LV-BRCA1 (MOI = 10) before OGD treatment. GFP immunofluorescence and protein expression of BRCA1 were detected to confirm transfection efficiency.

The transfected NSCs were transplanted using a 10 *μ*l Hamilton syringe 6 h after reperfusion. NSCs were prepared as single cell suspension in HBSS (1 × 10^5^cell/*μ*l). Cells were in situ given into the mouse cortex along the anterior/posterior (A/P) axis at three coordinates according to a previous report (2 *μ*l per coordinate) [16]. The coordinates are as follows: (1) A/P, +1.0; medial/lateral (M/L), +2.0; and dorsal/ventral (D/V), −1.0; (2) A/P, −0.5; M/L, +2.5; and D/V, −1.0; and (3) A/P, −2.0; M/L, +2.5; and D/V, −1.0. Cells were injected at 0.5 *μ*l/min, and the needle was left for 5 min post-injection before it was slowly removed.

### 2.5. Immunofluorescence

The mice were anesthetized and perfused with PBS followed by 4% paraformaldehyde (PFA) at indicated times. After postfixed in 4% PFA for 6 h, the brains were dehydrated in sucrose (10%, 20%, and 30%) and embedded in Tissue-Tek OCT compound (Sakura® Finetek USA). Then, the brains were cut into 25 *μ*m thick sections for histological staining. The brain sections and stem cell coverslips were fixed with 4% PFA for 20 min, followed by blocking with a solution containing 0.3% Triton, 3% goat serum, and 1% BSA. Subsequently, samples were incubated with primary antibodies against BRCA1 (1 : 200, Abcam, UK), GFP (1 : 500, Cell Signaling Technology, USA), BrdU (1 : 200, Abcam, UK), Nestin (1 : 200, Sigma-Aldrich, USA), *β*III-tubulin (1 : 400, Cell Signaling Technology, USA), GFAP (1 : 400, Cell Signaling Technology, USA), and CNPase (1 : 400, Abcam, UK) overnight at 4°C. After being washed with PBS for three times, the samples were incubated with appropriate secondary antibodies and 4′,6-diamidino-2-phenylindole (DAPI, Sigma-Aldrich, USA). Images were captured by FLUOVIEW FV1000 confocal microscopy (Olympus, Japan).

### 2.6. Cell Viability, Cell Death Assay, and BrdU Administration

Cell viability was assessed with Cell Counting Kit-8 assay kit (CCK-8; Dojindo, Japan) at indicated time points. Cell death was quantified by propidium iodide (PI)/Hoechst 33342 staining (Thermo Fisher Scientific, USA). For BrdU administration, the cells were treated with 10 mM BrdU for 24 h, and then, cell coverslips were incubated with anti-GFP (1 : 500, Cell Signaling Technology, USA) and anti-BrdU (1 : 200, Abcam, UK).

### 2.7. Superoxide Anion Detection and TUNEL Assay

Dihydroethidium (hydroethidine, DHE) staining was performed to detect the production of superoxide anions of cultured NSCs or grafted NSCs. *In vitro*, NSCs were incubated with 1 *μ*M DHE (Beyotime, China) in a culture medium for 15 min at 37°C. After being washed with PBS, DHE signals were captured with a BX71 fluorescence microscope (Olympus, Japan) and analyzed with Image J software (NIH, USA). *In vivo*, 200 *μ*l of DHE solution (1 mg/ml, Thermo Fisher Scientific, USA) was intravenously administrated after NSCs transplantation. 2 d after treatment, the mice were killed and brain sections were prepared. DHE and GFP double staining was carried out, followed by labeling with DAPI.

For cultured NSCs, cell apoptosis was assessed by TUNEL-AP (Millipore, USA) staining according to the manufacturer's instructions. For transplanted NSCs, cell apoptosis was detected by one-step TUNEL Apoptosis Assay Kit (Beyotime, China) 2 d after transplantation. Then, the sections were further incubated with anti-GFP (1 : 500, Abcam, UK) and Alexa Fluor 488-conjugated donkey anti-mouse IgG (1 : 400, Jackson ImmunoResearch, USA). TUNEL-positive GFP NSCs were counted using unbiased computational stereology as previously described [17].

### 2.8. Behavioral Testing

Modified neurological severity score (mNSS), an 18-point scoring system, which contains motor, sensory, balance, and reflex assessments, was utilized to evaluate sensorimotor deficits [18]. Adhesive-removal somatosensory test was utilized to assess somatosensory deficits [19]. Two small pieces of adhesive-backed paper dots were used. The time to remove each piece from the forelimb was recorded for four trials per day. Both behavioral tests were measured before MCAO surgery and on days 3, 7, 14, 21, and 28 after MCAO.

### 2.9. Quantification of Survival of GFP-Positive Transplanted NSCs

The transplanted GFP-positive cells were counted on day 28 post-MCAO using unbiased computational stereology as described previously [17]. The brain sections were co-immunostained with GFP and DAPI. GFP-positive cells were counted on five serial coronal sections per mouse brain (bregma, -2 mm to +2 mm).

### 2.10. Coimmunoprecipitation and GST Pull-Down Assay

Total cell lysates from OGD-treated NSCs were extracted using ice-cold RIPA lysis buffer (Cell Signaling Technology, USA). The proteins (500 *μ*g) were incubated with rabbit anti-BRCA1 (4 *μ*g, Abcam, UK), mouse anti-p53 (4 *μ*g, Abcam, UK), or control IgG for 12 h at 4°C with shaking, followed by linking to 40 μl of protein A/G-agarose beads (Cell Signaling Technology, USA) for another 4 h at 4°C. The beads were washed and denatured. Proteins were collected and analyzed by immunoblotting.

GST-BRCA1 proteins, including GST-BRCA1-1 (aa 15-172), GST-BRCA1-2 (aa 342-503), and GST-BRCA1-3 (aa 1591-1784), were constructed using pGEX-GST vectors and expressed in *E. coli* Rosetta (DE3) cells according to our previous methods [10]. The GST fusion proteins were purified using glutathione-Sepharose 4B beads. Then, total lysates of OGD/R-treated NSCs were added to the mixtures of GST fusion proteins and Sepharose beads for 4 h at 4°C. The beads were extensively washed with immunoprecipitation buffer and analyzed by immunoblotting with anti-GST (1 : 5000, Cell Signaling Technology, USA) and anti-p53 (1 : 1000, Abcam, UK).

### 2.11. Western Blotting

Brain tissues were harvested 2 d after transplantation, and cultured NSCs were harvested 24 h after reoxygenation. Samples were extracted by RIPA lysis buffer (Cell Signaling Technology, USA) to obtain whole-cell lysates. Protein concentrations were quantified by BCA protein assay kit (Beyotime, China). Equal amount of proteins were loaded and analyzed by SDS-PAGE gels and then probed with antibodies recognizing BRCA1 (1 : 1000, Abcam, UK), cleaved caspase-3 (1 : 1000, Cell Signaling Technology, USA), p53 (1 : 1000, Abcam, UK), Bax (1 : 1000, Cell Signaling Technology, USA), Bcl-2 (1 : 1000, Abcam, UK), NRF2 (1 : 1000, Abcam, UK), HO-1 (1 : 400, Santa Cruz Biotechnology, USA), NQO1 (1 : 1000, Abcam, UK), ubiquitin (1 : 3000, Abcam, UK), BDNF (1 : 1000, Abcam, UK), NGF (1 : 1000, Abcam, UK), or *β*-actin (1 : 1000, Cell Signaling Technology, USA). After incubating with appropriate HRP-conjugated secondary antibodies for 1 h, protein signals were detected by Immobilon Western Chemiluminescent HRP substrate (Millipore, USA) and were quantified by Image J software (NIH, USA).

### 2.12. Statistical Analysis

SPSS 22.0 software (IBM, Armonk, NY, USA) was used for data analysis. Results are presented as mean ± SD. Statistical comparisons were achieved using the Student *t*-test or one-way ANOVA followed by Tukey's post hoc analysis. Behavioral tests results were analyzed by repeated measures ANOVA followed by Tukey's *post hoc* test. Significance was accepted with *p* < 0.05.

## 3. Results

### 3.1. OGD/R Induced NSCs Apoptosis and BRCA1 Upregulation

**Figure 1:**
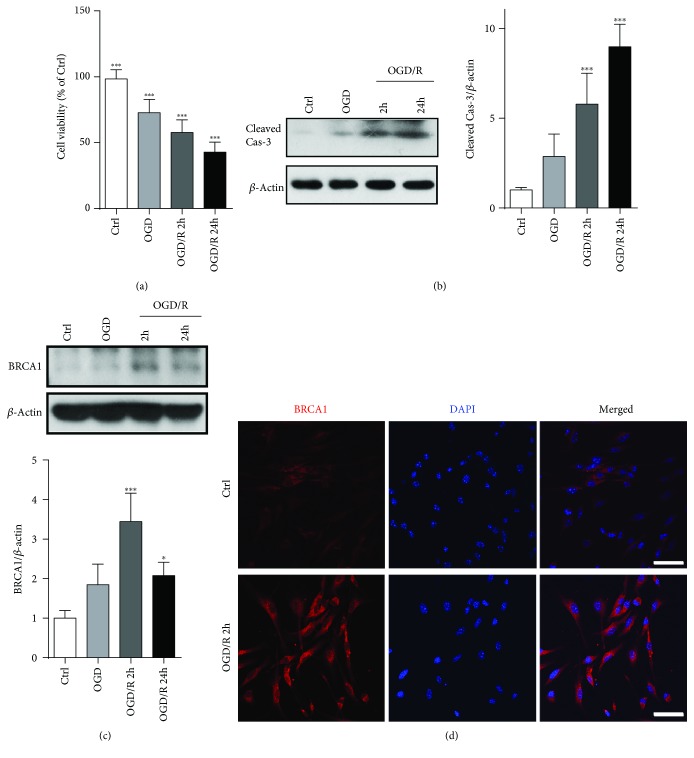
OGD/R induced NSCs apoptosis and triggered BRCA1 expression. The cultured NSCs were subjected to OGD for 4 h and subsequent reoxygenation for 2 h or 24 h. (a) Cell viability at the indicated times was detected by CCK-8. (b, c) Western blot and quantitative analysis of cleaved caspase-3 and BRCA1 in treated NSCs. (d) Immunofluorescence staining of BRCA1 in control and OGD/R NSCs. Data are expressed as mean ± SD; *n* = 5. ^∗^ *p* < 0.05; ^∗∗∗^ *p* < 0.001 vs. the control group. Scale bar: 50 *μ*m.

As shown in Supplementary Figure S1, up to 95% of cells were positive for Nestin, a marker of NSCs, through immunofluorescent staining. These cells differentiated into neurons, astrocytes, and oligodendrocytes (Supplementary Figure S1). CCK-8 assay demonstrated that cell viability decreased from 98.4 ± 6.9% to 72.7 ± 10.2% after OGD treatment; and it dropped to 42.8 ± 7.6% 24 h after reoxygenation (Figure 1(a)). An 8.9-fold upregulation of cleaved caspase-3 was detected 24 h after reoxygenation (Figure 1(b)). These data indicated that OGD/R administration induced NSCs apoptosis.

Meanwhile, OGD/R administration increased BRCA1 expression, with a peak at 2 h after reoxygenation and declined to 2.1-fold of control 24 h after reoxygenation (Figure 1(c)). Immunofluorescence analysis also confirmed the elevated BRCA1 signals (Figure 1(d)).

### 3.2. Overexpression of BRCA1 Reduced NSCs Apoptosis under OGD/R Condition

**Figure 2:**
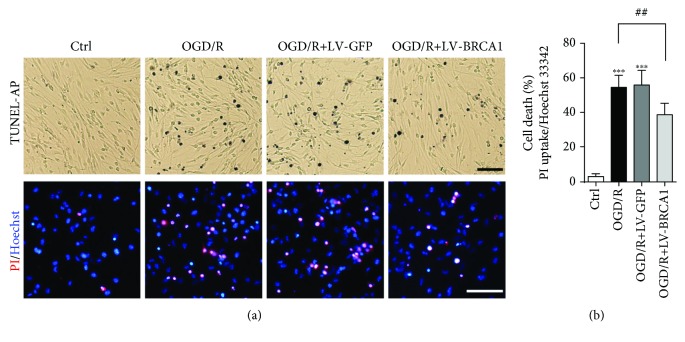
Overexpression of BRCA1 reduced NSCs apoptosis after OGD/R. NSCs were transfected with LV-GFP or LV-BRCA1 before OGD administration. (a) TUNEL-AP staining and PI/Hoechst 333342 double staining were performed to detect cell apoptosis 24 h after reoxygenation. (b) Quantification of PI-positive NSCs. OGD/R administration increased apoptosis of NSCs, which was attenuated by LV-BRCA1 transfection. Data are expressed as mean ± SD; *n* = 5. ^∗∗∗^ *p* < 0.001 vs. the control group; ^##^ *p* < 0.01 vs. the OGD/R group. Scale bar: 50 *μ*m.

To explore the role of BRCA1 in OGD/R-induced apoptosis of NSCs, LV-BRCA1-transfected NSCs were subjected to OGD/R. Western blotting detected a 4.7-fold expression of BRCA1 in LV-BRCA1-transfected NSCs than that of controls (Supplementary Figure S2). TUNEL-AP and PI/Hoechst 33342 staining demonstrated that OGD/R-induced NSCs apoptosis was significantly abolished by LV-BRCA1 transfection (Figures 2(a) and 2(b), *p* = 0.002).

### 3.3. BRCA1 Interacted with p53 and Inhibited p53-Mediated Proapoptotic Pathway

**Figure 3:**
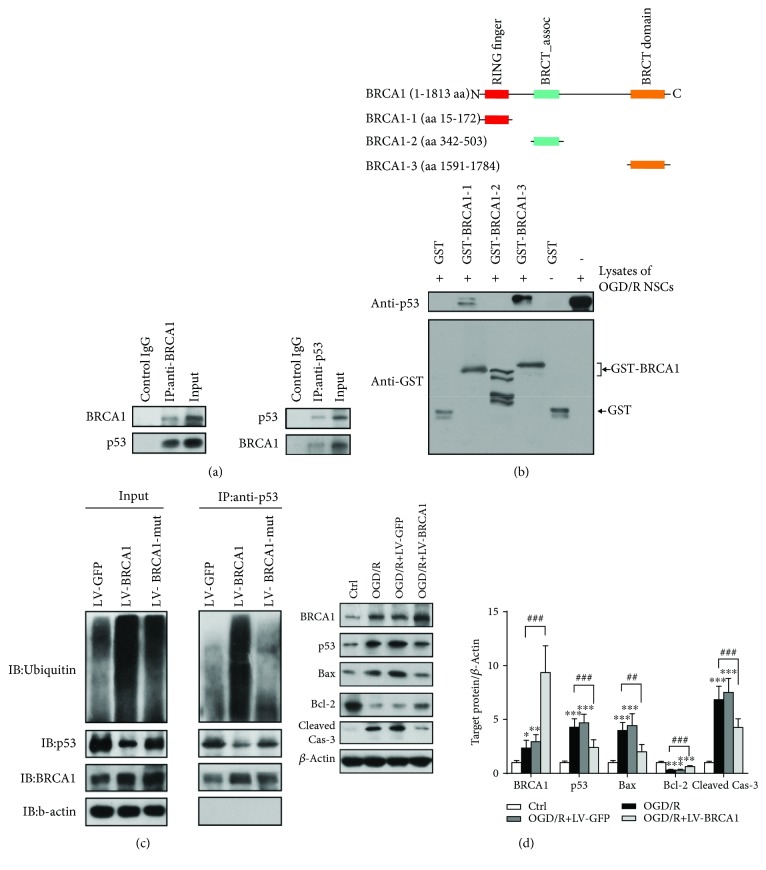
BRCA1 interacted with p53 and suppressed p53-associated apoptotic pathway. (a) The lysates from OGD/R-treated NSCs were immunoprecipitated with anti-BRCA1 or anti-p53. Then, total lysates and immunoprecipitates were subjected to immunoblot analysis. *n* = 3. (b) GST-BRCA1 fusion proteins containing different domains of BRCA1 were purified and incubated with OGD/R-treated NSC lysates. Protein to protein interaction was detected between RING finger domain and p53 as well as BRCT domain and p53. *n* = 3. (c) Transfected OGD/R NSCs were pretreated with 50 *μ*M MG132 for 6 h, and then, cell lysates were immunoprecipitated with anti-p53 antibody. The level of p53 ubiquitination was analyzed by western blotting. *n* = 3. (d) Western blot and quantitative analysis of p53 proapoptotic pathway 24 h after reoxygenation. Abolished upregulation of p53, Bax, and cleaved caspase-3 by OGD/R induction and reversed decrement of Bcl-2 by OGD/R induction were observed in BRCA1-overexpressed NSCs. *n* = 5. Data are expressed as mean ± SD; ^∗^ *p* < 0.05, ^∗∗^ *p* < 0.01, and ^∗∗∗^ *p* < 0.001 vs. the control group; ^##^ *p* < 0.01 and ^###^ *p* < 0.001 vs. the OGD/R group.

We here focused on the effects of BRCA1 on p53 proapoptotic pathway in OGD/R NSCs. BRCA1 interacted with p53 in OGD/R-treated NSCs though co-immunoprecipitation (Figure 3(a)). BRCA1 contains several functional domains that interact with different molecules [20]. To explore the binding domain of BRCA1 to p53, three GST-tagged proteins, including GST-BRCA1-1 (aa 15-172, RING finger domain), GST-BRCA1-2 (aa 342-503, BRCT_assoc domain), and GST-BRCA1-3 (aa 1591-1784, BRCT domain), were purified and incubated with lysates from OGD/R-treated NSCs (Figure 3(b)). We found that both the RING finger domain and BRCT domain could bind to p53 (Figure 3(b)). In addition, overexpression of BRCA1 increased ubiquitination of p53, but such results were not seen by transfection with LV-BRCA1-mut (RING finger domain deletion) (Figure 3(c)). OGD/R remarkably increased the levels of p53, Bax, and cleaved caspase-3, which were suppressed by LV-BRCA1 administration (Figure 3(d), *p* < 0.001, *p* = 0.004, and *p* < 0.001, respectively). Furthermore, LV-BRCA1 transfection significantly reversed the decrement of Bcl-2 by OGD/R (Figure 3(d), *p* < 0.001).

### 3.4. BRCA1 Promoted Cell Proliferation in OGD/R NSCs

**Figure 4:**
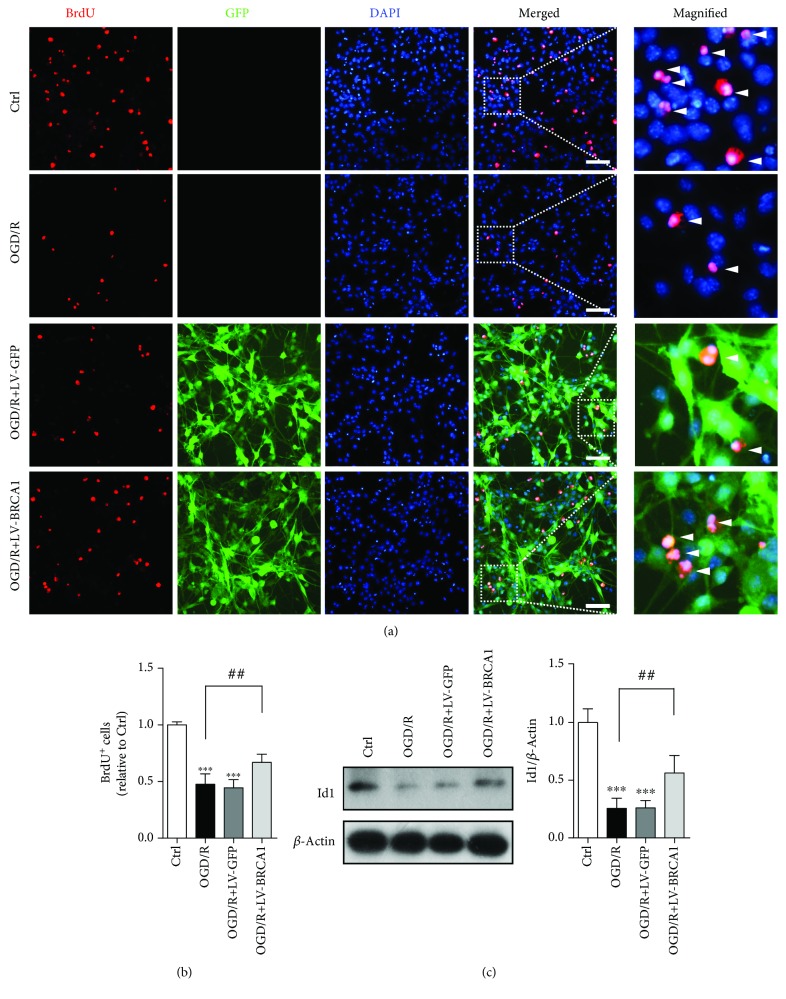
Overexpression of BRCA1 rescued cell proliferation in OGD/R NSCs. (a) BrdU/GFP immunostaining. (b) Quantification of BrdU-positive cells in the indicated groups. (c) Western blot and quantitative analysis of Id1 expression. Data are expressed as mean ± SD; *n* = 5. ^∗∗∗^ *p* < 0.001 vs. the control group; ^##^ *p* < 0.01 vs. the OGD/R group.

OGD/R reduced the proliferation of NSCs, which was rescued by LV-BRCA1 transfection (Figures 4(a) and 4(b), *p* = 0.003). A previous study reported that p53 represses the transcription of Id1, which promotes NSCs self-renewal [21]. Then, we tested whether BRCA1 could regulate Id1 expression. As shown in Figure 4(c), LV-BRCA1 transfection markedly ameliorated the decrement of Id1 protein expression in OGD/R NSCs (*p* = 0.002).

### 3.5. BRCA1 Mitigated Oxidative Stress in OGD/R NSCs

**Figure 5:**
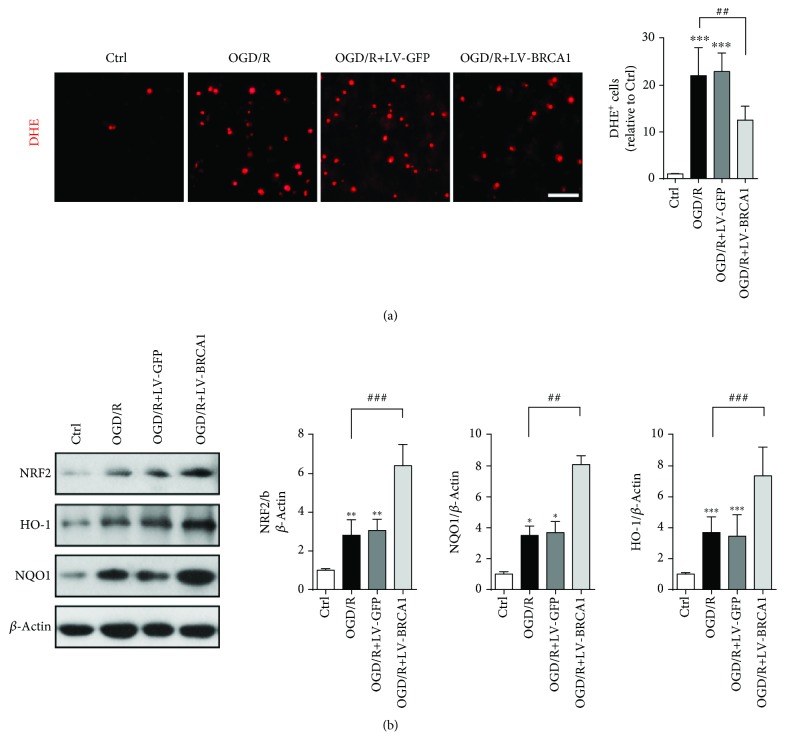
BRCA1 upregulation reduced oxidative stress in OGD/R NSCs. (a) DHE fluorescence staining was performed to detect intracellular ROS 24 h after reoxygenation. (b) Western blot and quantitative analysis of NRF2, HO-1, and NQO1 expression. Data are expressed as mean ± SD; *n* = 5. ^∗^ *p* < 0.05, ^∗∗^ *p* < 0.01, and ^∗∗∗^ *p* < 0.001 vs. the control group; ^##^ *p* < 0.01 and ^###^ *p* < 0.001 vs. the OGD/R group. Scale bar: 50 *μ*m.

OGD with subsequent 24 h reoxygenation caused a 22.1-fold increase in DHE-positive cells compared to controls, while LV-BRCA1 intervention significantly decreased the trends (Figure 5(a), *p* = 0.007). The upregulation of NRF2, HO-1, and NQO1 was amplified by LV-BRCA1 transfection against OGD/R (Figure 5(b), *p* < 0.001, *p* = 0.002, and *p* < 0.001, respectively).

### 3.6. BRCA1 Overexpression Reduced Grafted-Cell Apoptosis and ROS Production

**Figure 6:**
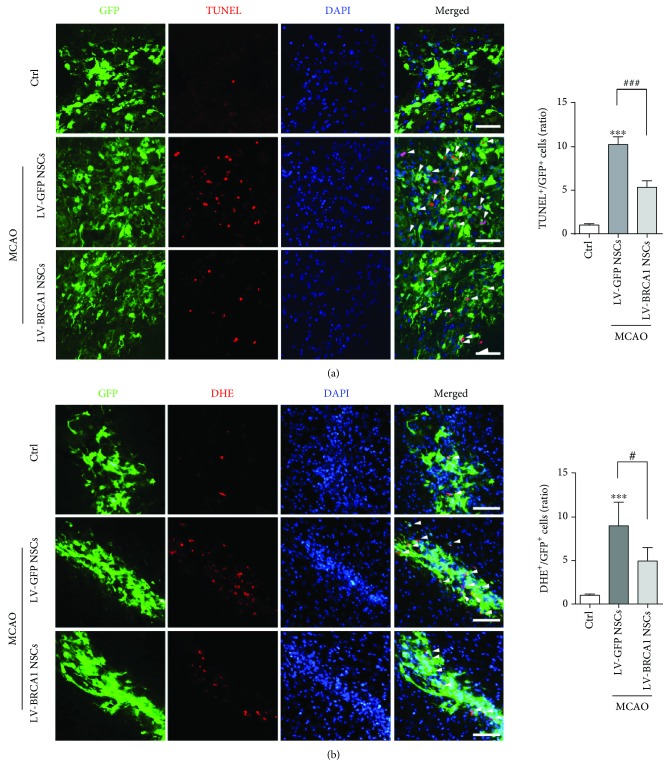
Reduced grafted cell apoptosis and oxidative stress by preincorporation of BRCA1 in NSCs. (a) 2 d after transplantation, immunostaining with GFP, TUNEL, and DAPI was performed. BRCA1 preincorporation significantly reduced the number of TUNEL-positive grafted cells. (b) Oxidative stress in grafted cells was elevated by immunostaining with GFP, DHE, and DAPI. BRCA1 overexpression markedly reduced DHE signals in implanted NSCs. Data are expressed as mean ± SD; *n* = 5 mice/group. ^∗∗∗^ *p* < 0.001 vs. the control mice; ^#^ *p* < 0.05 and ^###^ *p* < 0.001 vs. the LV-GFP NSCs-implanted mice. Scale bar: 50 *μ*m.

LV-GFP- and LV-BRCA1-transfected NSCs were implanted into the ischemic cortex 6 h after artery occlusion. At 2 d after transplantation, a 10.3-fold increase in TUNEL-positive grafted cells was seen in the brains, compared with control animals. And the trends were abolished by NSCs with BRCA1 overexpression (Figure 6(a), *p* < 0.001). Moreover, DHE signals in grafted cells increased noticeably than those in normal cells, which was alleviated by upregulation of BRCA1 (Figure 6(b), *p* = 0.011).

### 3.7. BRCA1 Manipulation Increased Trophic Factor Expression and the Survival of Grafted Cells

**Figure 7:**
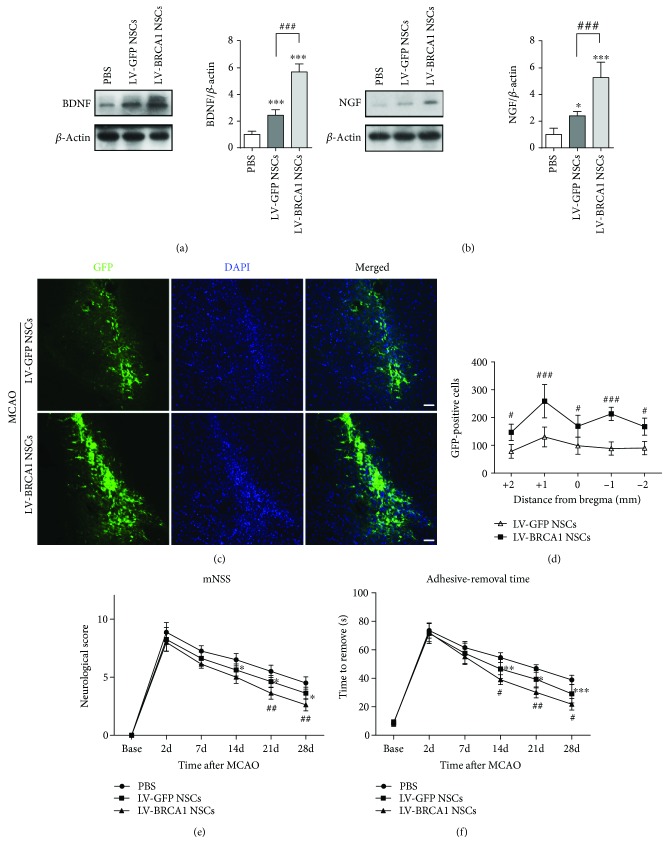
Modification with BRCA1 enhanced neurotropic factor expression, increased grafted cell survival, and improved neurobehavioral performance. (a, b) Western blot and quantitative analysis of BDNF and NGF in injured brain tissue 2 d after transplantation. *n* = 5 mice/group. (c) Representative images of GFP-positive NSCs that survived 28 d after stroke. Scale bar: 50 *μ*m. (d) Quantification of the number of surviving grafted cells. *n* = 5 mice/group. (e, f) The mNSS and adhesive-removal test were performed in MCAO mice. *n* = 8 mice/group. Data are expressed as mean ± SD; ^∗^ *p* < 0.05, ^∗∗^ *p* < 0.01, and ^∗∗∗^ *p* < 0.001 vs. the PBS-injected mice; ^#^ *p* < 0.05, ^##^ *p* < 0.01, and ^###^ *p* < 0.001 vs. the LV-GFP NSCs-implanted mice.

Transplantation with stem cells can provide trophic support to ischemic brain tissues by expressing neurotrophins [3]. We here tested the expression of BDNF and NGF 2 d after transplantation. The levels of BDNF and NGF were significantly increased in the LV-GFP NSCs-transplanted MCAO mice compared with the PBS-treated MCAO mice. BDNF and NGF expressions were further strengthened by modification of BRCA1 (Figures 7(a) and 7(b), *p* < 0.001 for both). The number of GFP-positive NSCs was counted 28 d post-stroke. More cells survived in the LV-BRCA1 NSCs than the LV-GFP NSCs after transplantation (Figures 7(c) and 7(d)).

### 3.8. Transplantation of BRCA1-Transfected NSCs Enhanced Neurological Functional Recovery

Neurological deficit performances were evaluated using the mNSS and adhesive-removal somatosensory test. As manifested in Figures 7(e) and 7(f), the MCAO mice with LV-GFP NSCs or LV-BRCA1 NSCs showed a lower mNSS score and less adhesive-removal time than the PBS-treated MCAO mice at 14, 21, and 28 d post-stroke. However, those mice with stroke with LV-BRCA1 NSCs displayed a reduced mNSS score (on days 21 and 28 post-stroke) and adhesive-removal time (on days 14, 21, and 28 poststroke), compared with the LV-GFP NSCs transfection group.

## 4. Discussion

After transplantation, exposure of hypoxia/ischemia microenvironment could cause grafted cell apoptosis [22, 23]. Local NSCs in the subventricular zone and subgranular zone are damaged by I/R cascade [24, 25]. Our results discovered that I/R induced NSCs oxidative stress and apoptosis both *in vivo* and *in vitro*.

BRCA1 exerts effects in DNA damage repair, oxidative stress, apoptosis, and ubiquitination [20]. Our previous study revealed that BRCA1 could rescue neurons from cerebral I/R injury [10]. In this study, we found that BRCA1 was triggered by OGD/R. Overexpression of BRCA1 could reverse NSCs apoptosis from OGD/R condition. It is believed that BRCA1 deletion leads to p53-dependent apoptosis in spermatocytes, cardiomyocytes, and embryonic neural progenitors, and apoptosis could be blocked through p53 deletion [11, 26, 27]. Consistent with this notion, we discovered that BRCA1 upregulation significantly decreased the p53 level as well as the Bax to Bcl-2 ratio in OGD/R NSCs. It suggested the effects of BRCA1 against p53 proapoptotic pathway. Nevertheless, Zhang and his colleagues reported that BRCA1 enhances p53-induced apoptosis through p53's transcriptional activity in SW80 human colon cancer cells [28]. The contradictory roles of BRCA1 in the regulation of apoptosis may be attributed to the differences in cell types and stimuli. BRCA1 has two important domains, the N-terminus RING finger binding motif and C-terminus BRCT motifs [20]. Experiments on cancer cells reported that aa 224-500 and aa 1760-1863 of human BRCA1 could bind to p53 [28, 29]. The present study indicated that both RING finger domain (aa 15-172) and BRCT domain (aa 1591-1784) of BRCA1 protein could connect with p53 in OGD/R NSCs. The RING finger domain of BRCA1 possesses E3 ubiquitin ligase activity [30]. Several proteins work as the substrate for BRCA1 ubiquitin, such as estrogen receptor *α*, progesterone receptor, and histone H2A [31–33]. We manifested in the present study that overexpression of BRCA1 increased ubiquitination of p53 in OGD/R NSCs, while the effects were abrogated by RING finger domain deletion. It should be a possible explanation that the crosstalk between RING finger domain and p53 promotes p53 ubiquitination and degradation. Ischemia stimuli induced p53 nuclear accumulation and activated its targets, such as Bax, PUMA, and Noxa in neurons [34, 35]. Interestingly, BRCA1 ablation resulted in p53 nuclear translocation in neural progenitors [9]. We may speculate that BRCA1 influenced p53 nuclear translocation in NSCs post-OGD/R. But more studies need to be done in the future.

BRCA1 expression is linked to NSCs proliferation [8]. The BRCA1 exon 5-6 mutant in embryos notably impaired cellular proliferative capability [12]. NSCs proliferation was restored by LV-BRCA1 transfection against OGD/R. It is reported that p53 deficiency upregulates Id1 through BMP-Smad1-dependent and independent ways, resulting in NSCs proliferation and neuronal differentiation [21]. We showed that BRCA1 repressed the level of p53 and enhanced the level of Id1, indicating an axis from BRCA1 to p53-Id1.

Oxidative stress induced by ischemia leads to massive loss of grafted NSCs [15, 36]. Improving the antioxidative capability of grated NSCs may ensure the survival ability. BRCA1 could repress oxidative stress in I/R-injured neurons through NRF2/ARE pathway [10]. As a key cellular stress resistance factor, NRF2 influences NSCs survival and function [37, 38]. Our previous study indicated that NRF2 is a direct target of BRCA1 [10]. Indeed, using LV-BRCA1, we also demonstrated that BRCA1 activated the NRF2/ARE antioxidant pathway and subsequently reduced ROS production in OGD/R NSCs.

Our findings revealed that pre-incorporation of BRCA1 into NSCs improved cell survival and induced greater neurofunctional recovery. Though clinical trials have manifested the safety and feasibility of neural stem cell implantation [39, 40], there are no clinical trials using gene-modified stem cells for stroke treatment. Additional works are needed to elevate the safety and efficacy of implanting gene-modified cells into patients.

In conclusion, we reported that gene overexpression of BRCA1 in NSCs reduced cell apoptosis and oxidative stress and promoted cell proliferation post-OGD/R. Modification of BRCA1 could enhance the effectiveness of NSCs transplantation in ischemic stroke.

## Data Availability

The data used to support the findings of this study are available from the corresponding author upon request.

## Abbreviations

ARE: Antioxidant response element
BRCA1: Breast cancer susceptibility protein 1
BDNF: Brain-derived neurotropic factor
bFGF: Basic fibroblast growth factor
CCK-8: Cell Counting Kit-8
DAPI: 4′,6-Diamidino-2-phenylindole
DMEM: Dulbecco's modified Eagle medium
DHE: Dihydroethidium
ECA: External carotid artery
EGF: Epidermal growth factor
HBSS: Hank's balanced salt solution
HO-1: Heme oxygenase 1
ICA: Internal carotid artery
I/R: Ischemia/reperfusion
ITSS: Insulin-transferrin-sodium selenite supplement
MCAO: Middle cerebral artery occlusion
mNSS: Modified neurological severity score
NRF2: Nuclear factor (erythroid-derived 2)-like 2
NGF: Nerve growth factor
NPCs: Neural precursor cells
NQO1: NAD(P)H quinone dehydrogenase 1
NSCs: Neural stem cells
OGD/R: Oxygen-glucose deprivation/reoxygenation
PI: Propidium iodide
ROS: Reactive oxygen species
TUNEL: Terminal deoxynucleotidyl transferase-mediated dUTP nick end labeling.

## Supplementary Materials

Supplementary Materials Figure S1: (A) Immunofluorescence identification of NSCs with anti-Nestin. (B) Fluorescence images of differentiation-induced NSCs stained with anti-*β*III tubulin (neuron marker), anti-GFAP (astrocyte marker), or anti-CNPase (oligodendrocyte marker). Scale bar: 50 *μ*m. Figure S2: Transfection efficiency of LV-BRCA1 was detected in NSCs. (A, B) GFP-positive transfected NSCs in the LV-GFP and LV-BRCA1 group with quantification. (B) Western blot and quantitative analysis of BRCA1 expression 72 h after transfection. Data are expressed as mean ± SD; *n* = 5. ^∗∗∗^ *p* < 0.001 vs. the control group.
