# Supplementary material for: Overexpression of BRCA1 in Neural Stem Cells Enhances Cell Survival and Functional Recovery after Transplantation into Experimental Ischemic Stroke
Source: Oxid Med Cell Longev. 2019 Apr 3;2019:8739730. doi: 10.1155/2019/8739730 (PMC6470423; doi:10.1155/2019/8739730)
Supplement: Supplementary Materials — Figure S1: (A) Immunofluorescence identification of NSCs with anti-Nestin. (B) Fluorescence images of differentiation-induced NSCs stained with anti-βIII tubulin (neuron marker), anti-GFAP (astrocyte marker), or anti-CNPase (oligodendrocyte marker). Scale bar: 50 μm. Figure S2: Transfection efficiency of LV-BRCA1 was detected in NSCs. (A, B) GFP-positive transfected NSCs in the LV-GFP and LV-BRCA1 group with quantification. (B) Western blot and quantitative analysis of BRCA1 expression 72 h after transfection. Data are expressed as mean ± SD; n = 5. ∗∗∗ p < 0.001 vs. the control group. [file 8739730.f1.pdf]

Overexpression of BRCA1 in neural stem cells enhances cell survival and functional recovery after transplantation into experimental ischemic stroke

Pengfei Xu[a,#], Xiaolei Shi[b,c,#], Xiaohao Zhang[d,#], Qian Liu[a], Yi Xie[a], Ye Hong[a], Juanji Li[a], Mengna Peng[a], Xinfeng Liu[a], Gelin Xu[a]

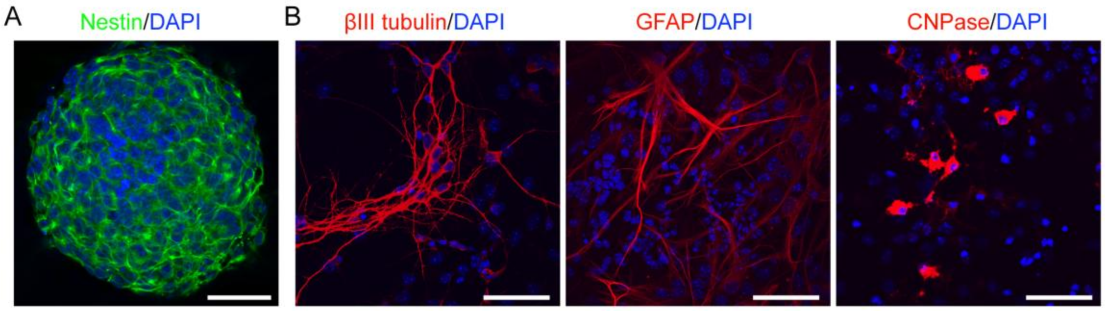

Figure S1 (A) Immunofluorescence identification of NSCs with anti-Nestin. (B) Fluorescence images of differentiation-induced NSCs stained with anti-βIII tubulin (neuron marker), anti-GFAP (astrocyte marker), or anti-CNPase (oligodendrocyte marker). Scale bar: 50 μm.

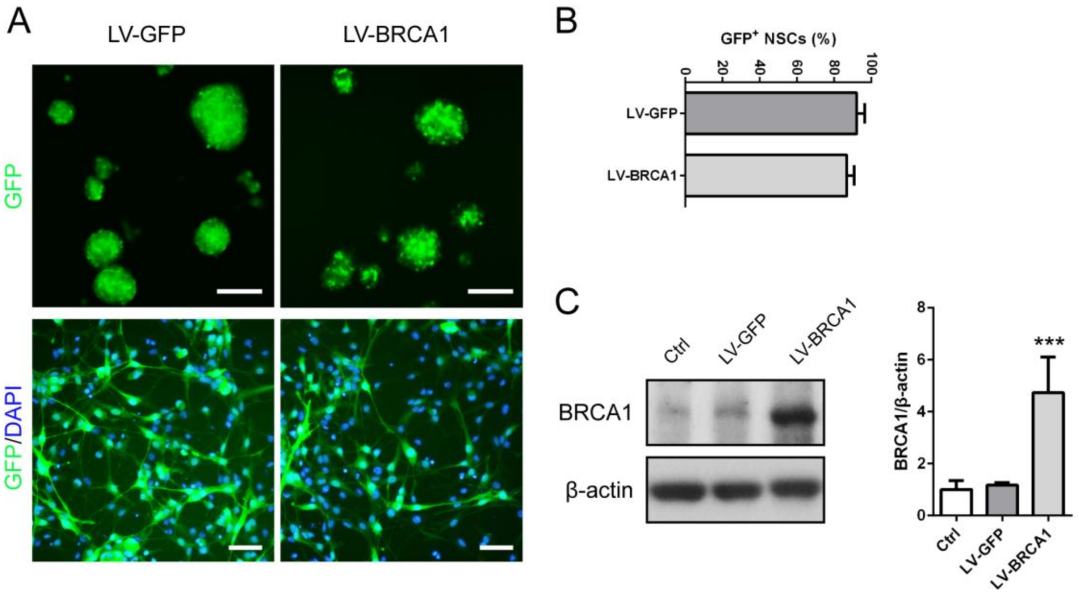

Figure S2 Transfection efficiency of LV-BRCA1was detected in NSCs.

(A, B) GFP-positive transfected NSCs in LV-GFP and LV-BRCA1 group with quantification. (B) Western blot and quantitative analysis of BRCA1 72 h after transfection. Data are expressed as mean ± SD; n = 5. ***p < 0.001 vs control group.
